# Evaluating an organization-wide disparity reduction program: Understanding what works for whom and why

**DOI:** 10.1371/journal.pone.0193179

**Published:** 2018-03-14

**Authors:** Sivan Spitzer-Shohat, Efrat Shadmi, Margalit Goldfracht, Calanit Key, Moshe Hoshen, Ran D. Balicer

**Affiliations:** 1 Department of Population Health, Azrieli Faculty of Medicine, Bar-Ilan University, Safed, Israel; 2 Center for Health and Social Sciences, University of Chicago, Chicago, IL, United States of America; 3 Faculty of Social Welfare and Health Sciences, University of Haifa, Haifa, Israel; 4 Clalit Research Institute, Chief Physician’s Office, Clalit Health Services, Tel Aviv, Israel; 5 Clalit Community Division, Clalit Health Services, Tel Aviv, Israel; 6 Public Health Department, Faculty of Health Sciences, Ben-Gurion University, Beer-Sheva, Israel; University of Kentucky, UNITED STATES

## Abstract

**Background:**

Disparity-reduction programs have been shown to vary in the degree to which they achieve their goal; yet the causes of these variations is rarely studied. We investigated a broad-scale program in Israel’s largest health plan, aimed at reducing disparities in socially disadvantaged groups using a composite measure of seven health and health care indicators.

**Methods:**

A realistic evaluation was conducted to evaluate the program in 26 clinics and their associated managerial levels. First, we performed interviews with key stakeholders and an ethnographic observation of a regional meeting to derive the underlying program theory. Next, semi-structured interviews with 109 clinic teams, subregional headquarters, and regional headquarters personnel were conducted. Social network analysis was performed to derive measures of team interrelations. Perceived team effectiveness (TE) and clinic characteristics were assessed to elicit contextual characteristics. Interventions implemented by clinics were identified from interviews and coded according to the mechanisms each clinic employed. Assessment of each clinic’s performance on the seven-indicator composite measure was conducted at baseline and after 3 years. Finally, we reviewed different context-mechanism-outcome (CMO) configurations to understand what works to reduce disparity, and under what circumstances.

**Results:**

Clinics’ inner contextual characteristics varied in both network density and perceived TE. Successful CMO configurations included 1) highly dense clinic teams having high perceived TE, only a small gap to minimize, and employing a wide range of interventions; (2) clinics with a large gap to minimize with high clinic density and high perceived TE, focusing efforts on tailoring services to their enrollees; and (3) clinics having medium to low density and perceived TE, and strong middle-management support.

**Conclusions:**

Clinics that achieved disparity reduction had high clinic density, close ties with middle management, and tailored interventions to the unique needs of the populations they serve.

## Introduction

Disparity reduction efforts, focusing on minimizing differences in health and health care, have been the focus of health care systems for over 30 years[1]. Although evidence of success is accumulating[2–4], programs vary in how well they achieve their goals, with some even showing increased disparities over time[5,6]. To better understand how success is achieved, recent research calls for in-depth investigation of how programs work, evaluating the factors associated with success on a variety of outcomes, and understanding the processes that lead to attainment of disparities reduction[1,7–9].

Israel is an example of a society in which, despite the availability of national health insurance since 1995[10], gaps are evident in a wide range of indicators, such as life expectancy, infant mortality, prevalence of chronic diseases, and use of health care services[11–17]. To address these expanding gaps, in 2009 Clalit Health Services, Israel’s largest non-for-profit insurer and provider of services, covering about 54% of the population, initiated an organization-wide program to reduce gaps between low-SES and minority populations and its general member population [2]. The program targeted 55 primary care clinics serving approximately 400,000 people (10% of Clalit’s population), of mainly economically disadvantaged and minority groups, who were identified as performing poorly on a composite measure of seven health and health care indicators (the Quality Indicator Disparity Scale—QUIDS): diabetes, hypertension, and lipid control; prevention of anemia in infants; mammography screening; fecal occult blood tests; and influenza vaccinations. The aim of the program was to close the gap between target clinics and all other comparable clinics (medium to large, serving 5,000–15,000 members). Although the overall organizational goals for disparity reduction and the measurement scheme were set by the central Clalit management, the interventions formulated and their implementation strategy were developed locally at the regional, subregional, and primary-care clinic levels.

Between 2009 and 2012 a significant decrease in the gap, based on the composite quality measure, was reported[2,18]. However, clinics differed significantly in their outcomes, eliciting the need to better understand the sources of these variations[19]. To address this need, we performed a Realist Evaluation (RE), to identify ‘what works in which circumstances and for whom?’ [20]. We assessed contextual drivers, including clinic team perceptions on possessing the necessary resources and skills to succeed (perceived team effectiveness [TE]), as well as relationships among team members and with their superior managerial units. We also examined the types of interventions employed by each of the participating clinics, and the relation between the unique organizational context, the types of interventions implemented, and the attainment of the organizational disparity-reduction goal.

## Methods

### Setting

The program was initiated and carried out in Clalit, that operates its integrative health-care delivery system through a decentralized structure comprised of a central management and eight semi-independent geographic regional management units spread throughout the country. Each region manages its own budget, funded through capitated payment, and provides all primary and specialty care, imaging, lab, and pharmacy services to its member population. Within each region, the regional headquarters oversees 3 to 4 subregional management in charge of all the services in that subregion, including 5 to 10 primary care clinics, operating similarly to Accountable Care Organizations (ACOs) [21]. Primary care clinics are organized in teams of physicians, nurses, pharmacists, and administrative employees. Since 2002 all health care encounters in Clalit are electronically registered and data are stored and managed in a central data warehouse, through which financial, clinical, and health-care-quality performance is assessed [22].

### Research strategy

We employed an RE framework, identifying the context in which each program operates, the mechanisms through which it is implemented, and its outcome, to form context-mechanism-outcome (CMO) configurations [23–26]. Data were collected using a retrospective cross-sectional mixed-methods sequential explanatory design (qualitative and quantitative)[27]. We evaluated disparity-reduction program implementation in 26 of the 55 target clinics and their associated managerial levels, which serve 217,306 enrollees in four of the organization’s eight geographic regions. Clinics were selected (from the pool of 55), to ensure representation of the characteristics of the focus clinics’ population such as rural (n = 19) and urban (n = 7) areas, and representation of patients’ ethnicity and cultural including Jewish ultra-orthodox communities (n = 2) and low-socio economic peripheral communities (n = 4) and Arab minorities (n = 20) [2]. Analysis was carried out in three phases: (1) identifying the relevant program theory through key stakeholders, (2) collecting data and analyzing program implementation, and (3) proposing CMO configurations to explain the observed variations in target clinics’ success in reducing disparity.

#### Phase 1: Identifying program theory of disparity reduction

In line with the realist methodology [20], as a first step, we interviewed key informants (n = 6) from all managerial levels in the organization: head of the disparity-reduction program at Clalit headquarters, head of the organization’s quality unit, a regional headquarters director, a regional headquarters quality director, a subregional medical director, and a clinic director. Interviews focused on eliciting information about program implementation and on understanding the organizational framework for action. An ethnographic observation of a regional meeting that included inter-professional managerial-team members of target clinics: nursing, medical, administrative, and pharmacy personnel from the regional, subregional, and clinic teams, was also performed. We observed the following domains: priorities set by clinics during the presentation, the make-up of the presenting team, the type of discourse with the managerial level (e.g., defensive, open, oriented towards problem solving), and the types of solutions and directives provided by the management (e.g., focused, integrative, geared towards sustainability).

Data, including observational notes and transcriptions of interviews held with key informants, were analyzed, eliciting themes relating to both the overall program theory underpinning the disparities reduction program as well as strategies for action. Plausible strategies, that is, mid-range theories, motivating the focus of program implementation were identified. Meetings were held with study team members to discuss emerging themes, which were then refined based on feedback and discussion to strengthen validity [19,28].

#### Phase II: Data sources and analysis of the disparity-reduction program

**Fig 1:**
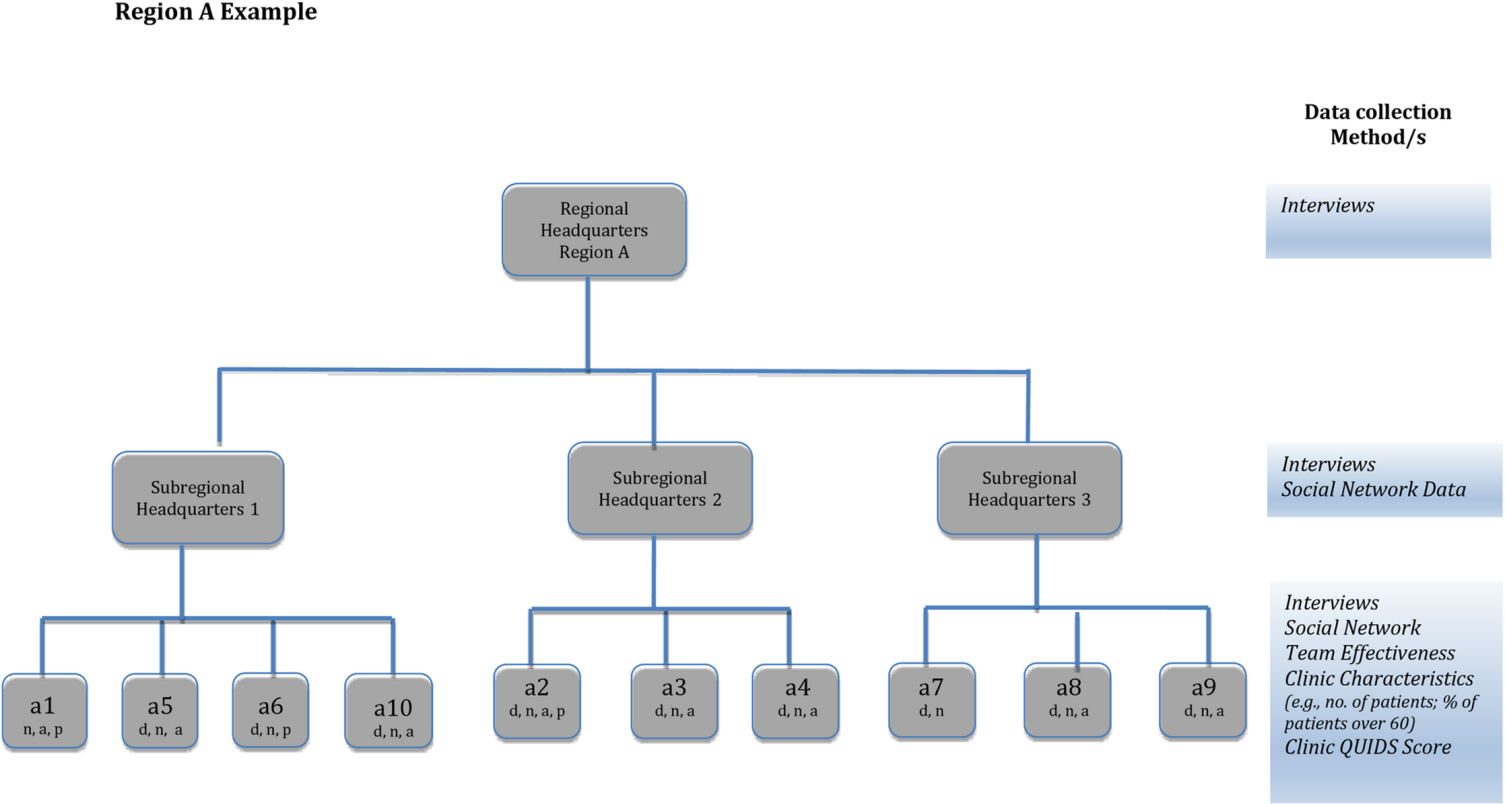
Outline of organizational structure and data collection strategy. d = clinic director, a physician; n = nursing director; a = administrative head; p = pharmacist.

To capture the complex and dynamic nature of the disparity-reduction effort, we drew on a wide range of data sources, methods, and materials [28,29], including in-depth semi structured interviews with all stakeholders at all managerial levels, social network analysis (SNA) of data on the relationships between all actors participating in the program, the clinic teams' perceptions of their ability to implement the program (TE), data on clinic characteristics such as size and patient population served, and QUIDS scores at baseline and 3-year follow-up. The data sources are presented in Fig 1 and outlined below.

#### Qualitative data sources and analysis

Interviews were conducted between July 2010- May 2011 by two of the authors. To elicit a wide variety of perspectives [30], we interviewed 80 inter-professional managerial-team members, including medical directors, nursing directors, heads of administration, and pharmacists (in clinics with an in-house pharmacy) in each of the 26 clinics; 19 medical and nursing directors in 10 subregional headquarters; 8 medical and nursing regional headquarter directors; and 2 quality-assurance directors. Verbal consent was obtained from interviewees.

The interview guide was adapted from the work of Chin and colleagues (2008) [31] and Wasserman and Faust (1994) [32], and then pilot-tested with key informants at five clinics. Interviews focused on (a) eliciting the perceptions of the stakeholders about what worked (or not) in achieving disparity reduction, and (b) understanding the organizational processes by probing issues such as the day-to-day relations among clinic team members, the relationship between clinic teams and their respective managerial units, and the relationship with the community of the patient population they serve. Questions also aimed to identify what interventions were implemented to address each of the seven QUIDS indicators and to explore participants’ reasoning and resources, that is, the mechanisms underpinning the strategy selected [26].

Interviews were recorded and transcribed, and a key informant was identified for each clinic. The information provided by the key informant was augmented by information from other team members and from the relevant subregional managerial personnel. Upon completing the interviews, we performed a member check by sending summaries to each clinic, to validate the information elicited and to allow interviewees the opportunity to refine or add comments [33].

We mapped the mechanisms according to the program theory identified in phase I [19]. We coded the data based on emergent themes through an iterative process of reading and re-reading the interview transcripts. We conducted a thematic analysis to identify the strategies employed and interventions implemented [34]. We then rated the scope of each type of intervention as low, medium, or high, reflecting the degree to which the selected intervention was a priority and the extent of its implementation [35]. The coding technique was pilot-tested by two of the authors (SS and ES), who independently classified data from four clinics and then discussed their findings to reach consensus.

#### Quantitative data collection

Social network data were obtained through a questionnaire administered to clinic teams and subregional headquarters directors, which included a roster for identifying network ties. The roster listed the relevant organizational positions at the clinic, subregional, and regional levels. Respondents were asked to rate “To what extent do you consult with members of your organization on matters pertaining to the disparity reduction initiative?” [36]. Answers ranged from 1 (*very little*) to 5 (*a lot*). Data on the types and intensity of ties were used to generate matrices of ties between network actors (nodes) and to assess structural-network measures of density using a weighted measure (the sum values of all ties divided by the number of possible ties) of within clinic networks, clinic–subregional headquarters networks, clinic–regional headquarters networks, and overall regional networks (including all clinic, subregional, and regional teams) [32,37]. The measure of density reflects the number of actors in contact. The connection of actors in dense networks can provide social support to team members when they are implementing a new program [38,39].

Perceived Team Effectiveness (TE) was assessed using the validated team effectiveness instrument to determine whether clinic teams believed they had the necessary information, authority, and autonomy to implement the disparity-reduction program; whether they had the skills to implement the necessary changes; whether they agreed with the organization’s goal to minimize disparities and on how this should be achieved; and whether they felt supported by the organization in implementing the program S1 File [40]. A clinic composite perceived TE score was constructed based on the average rating of all participants in each of the 26 clinics. To enable the creation of CMO configurations according to perceived TE levels, we categorized clinics into tertiles as low-, medium-, and high-performing TE clinics based on the following cut points: 3.68 or below, 3.69 to 4.02, and above 4.02, respectively.

QUIDS performance data for each of the clinics, as well as contextual characteristics such as clinic size and SES indicator score rating all residential areas in Israel, from 1—low SES to 10 high SES, [41] were obtained from Clalit’s central data warehouse. We calculated the gap in QUIDS scores for each clinic versus all other comparable clinics in each region at baseline (2009) and at 3-year follow-up (2012). Disparity reduction was defined as the relative reduction in the gap in QUIDS scores between 2009 and 2012 for each participating clinic compared with the average score of all other clinics in its region. We categorized clinics according to the QUIDS gap at the beginning of the disparity-reduction program (small versus large gap) according to the following tertiles: small gap (< 0.06), medium gap (0.06–0.09), and large gap (>0.09).

#### Phase III: Constructing clinics’ context-mechanism-outcome configurations

CMO configurations were constructed through an iterative process, checked against collected data, and refined [19,42]. Reduction in the gap in QUIDS scores between 2009 and 2012 for each participating clinic was assessed against contextual characteristics of the clinics and the mechanisms they employed. Configurations were identified by assessing different patterns of change and their interplay with elicited mechanisms, and by comparing clinics that attained the organizational goal with clinics that did not.

The study protocol was approved by the Committee on Human Studies, Clalit Health Services (authorization no. 172/2011).

## Results

### Phase I: Analysis of key stakeholders’ perspectives

Analysis of stakeholder's interviewed revealed several themes that guided their approach towards disparity reduction, including: quality improvement and organizational performance. The stakeholders emphasized the uniqueness of Clalit’s disparity-reduction program in its aim to not only monitor but create a plan of action: “Other programs aim to improve overall quality while this program targets the clinics which are the lowest-performing and most challenged. To improve overall average performance, it is usually easier to work with the early adopters, those most receptive to change. Yet, the disparity-reduction program put the spotlight on those clinics in which it is most difficult to achieve change, forcing us and them to put extra effort to improve at a faster rate than the average rate of improvement” [Head, Disparity-Reduction Program, central management].

Management focused on performance measurement, and perceived clinics as context experts responsible of tailoring the program to their patient population: “As in similar organization-wide strategies, we only ‘set the stage’ by setting the goals and providing monitoring and feedback. In order to tailor the program for unique populations, it was clear that the improvement strategy should be designed by each clinic and supported by regional headquarters” [Head, Community Quality-Improvement Unit, central management].

Analysis of Interviews with a target clinic directors as well as ethnographic observation of a regional meeting showed of an existing similar discourse to that of central management in the division of responsibilities between measurement and implementation. Clinics combined knowledge of performance data with adapting and tailoring services to the clinic’s setting: “I looked at the micro-surrounding of the clinic and translated the way we work to fit our surrounding… only after we trained the clinic team on the meaning and measurement of each indicator [could we] think how to adapt it to our patients… This is the process that I see all successful clinics have adopted… working in a two-stage process of training the team and then adapting care to the patient population served, which is the most challenging task that we have” [Clinic Director].

Analysis of the discourse elicited in this phase highlighted the organization’s focus on reducing disparities mainly through the framework of quality improvement (QI), an outcome-driven approach centering on identifying a process with less than ideal outcomes, measuring key performance attributes, and devising a plan of action [43].

Utilizing QI as the guiding program theory, we searched for common intervention strategies. QI programs focus on implementing changes at the provider and/or patient level [1]. Provider-focused strategies aim to improve the organization’s work, and they emphasize internal processes such as teamwork, delivery of services, and data management [38,44]. Patient-level interventions often focus on providing patient-centered services; most of these interventions focus on educating patients about specific chronic conditions [45–47]. Hence, we elicited two main mechanisms: (A) a focus on the provider, i.e. improving day-to-day operations through teamwork, delivery-system design (such as improved access to specialists), and promoting effective use of the organization’s patient registry of computerized electronic health records; and (B) a focus on patients, aimed at patient education and at tailoring care processes and services to the needs of the specific patient population, taking into account cultural and religious values and beliefs, performing outreach and establishing linkages with community services/leaders. The two main mechanisms identified are mechanisms implemented by clinic teams. While the provider mechanisms focuses internally on improvement by clinic teams on the way in which the day to day work is implemented, the patient focused mechanisms focuses on the way in which clinic teams’ address external patient factors that impact their day to day work.

### Phase II: Analysis of target clinics’ characteristics

#### Context of target clinics

Table 1 details the contextual characteristics of each of the 26 clinics. We examined the characteristics of the clinics’ patient populations (outer context) as well as the clinics’ teams (inner context). Twenty clinics served enrollees of Arab minority populations; one clinic served an ultra-orthodox Jewish community (d1). Most target clinics were large, serving over 6,000 enrollees, from disadvantaged low-SES populations. Clinics with high SES scores all had relatively high percentages of enrollees over the age of 65 (a4, c1, c2, c3).

**Table 1:** Context: Characteristics of target clinics.

	Clinic-Patient characteristics (outer context)					Clinic-Organizational Context (inner context)			
	Serving Arab Minority Patients	Size of Clinic	Enrollees Average Age	% of Enrollees over 65	Socio-Economic Index	Clinic Density	Clinic Sub-regional headquarters density	Team Effectiveness Score	Team Effectiveness Level
**Region A**									
Clinic a1	+	3	33	13.63	4	3.75	0.82	3.96	2
Clinic a2	+	2	27	06.5	3	4.33	0.55	4.16	3
Clinic a3	+	2	28	012.5	3	2.67	0.38	3.54	1
Clinic a4		2	27	14.06	6	2.56	0.83	3.39	1
Clinic a5	+	2	26	7.35	2	3.58	1.00	3.69	2
Clinic a6	+	3	47	11.78	3	3.00	0.71	3.62	1
Clinic a7	+	2	30	4.53	3	2.17	1.05	4.28	3
Clinic a8	+	3	24	7.26	3	3.33	1.07	3.65	1
Clinic a9	+	3	35	10.15	3	4.50	1.43	3.43	1
Clinic a10	+	3	42	7.71	3	3.75	1.05	3.85	2
Region B									
Clinic b1		2	52	8.58	5	3.33	0.84	4.02	2
Clinic b2	+	3	32	7.07	2	2.92	1.09	4.33	3
Clinic b3	+	3	33	6.18	6	3.00	1.07	4.36	3
Clinic b4		3	28	38.08	4	3.75	1.16	4.44	3
Clinic b5	+	3	31	10.28	3	2.92	0.84	4.17	3
Region C									
Clinic c1	+	3	27	34.04	7	4.83	0.88	4.15	3
Clinic c2		3	25	36.76	7	3.25	0.41	3.83	2
Clinic c3		2	52	31.56	7	3.33	0.62	4.10	2
Region D									
Clinic d1		1	27	3.90	2	4.67	1.14	4.37	3
Clinic d2	+	3	27	13.08	4	2.92	0.86	3.98	2
Clinic d3	+	2	50	6.19	2	0.25	0.27	3.05	1
Clinic d4	+	3	22	9.10	4	0.92	0.38	3.42	1
Clinic d5	+	3	31	5.38	2	3.42	0.59	3.99	2
Clinic d6	+	3	28	7.49	3	3.25	0.98	3.89	2
Clinic d7	+	3	30	7.17	4	3.82	0.62	3.40	1
Clinic d8	+	3	46	16.71	3	2.92	0.86	3.98	2

Size of Clinic: 1 = up to 2000 enrollees; 2 = 2001–6000 enrollees 3 = over 6001 enrollees; Socio-Economic Index: 1 = low socio-economic status 10-high socio economic status; Team Effectiveness level 1 = 3.68 or below, 2 = 3.69 to 4.02, and 3 = 4.02 and up

The inner organizational characteristics of the clinics varied in terms of both network density and perceived TE. Clinics a2, c1, and d1 reported high inner clinic density as well as high levels of perceived TE. Clinics a3, a4, a8, a9, d3 and d4, on other hand, reported low inner clinic density and low perceived TE levels. Some clinics reported a mix of dense ties with subregional headquarters and low levels of TE (a5 and a9).

#### Mechanisms employed by target clinics

Table 2 demonstrates the two main mechanisms—focusing on the provider and focusing on the patient—elicited from thematic analysis of the interviews. Overall, clinics implemented 454 interventions, ranging from 9 (a7) to 29 (b3) (x = 17.5 sd±5.4). Clinics also differed in the mechanism adopted: 5 clinics concentrated on improving teamwork (a5, b1, b3, b4, b5), 8 concentrated on educating or improving care processes (a2, b1, b2, b3, b4, b5, c1, d1), and 5 focused on community outreach (a2, a8, a10, c1, d1). All clinics reported medium to high use of the organization’s patient registry to design proactive patient outreach as a preferred strategy for improving performance.

**Table 2:** Implementation of QI program by target clinics.

	No. of Interventions	Mechanisms Focused on the Provider			Mechanisms Focused on the Patient	
		Team Work	Delivery System (Accessibility)	Computerized Patient Registry	Patient Education and/or care plans	Community Linkages
**Region A**						
Clinic a1	22	++	++	+++	++	++
Clinic a2	25	+	+	+++	+++	+++
Clinic a3	11	+	+	+++		+
Clinic a4	12	+		+++	+	
Clinic a5	15	+++	++	+++	+	
Clinic a6	19	++	++	+++	++	++
Clinic a7	9	+	+	+++	+	+
Clinic a8	26	++	+	+++	+	+++
Clinic a9	16	++	+	+++		
Clinic a10	17	++	+	+++	++	+++
**Region B**						
Clinic b1	19	+++	+	++	+++	++
Clinic b2	24	++	+	++	+++	++
Clinic b3	29	+++	+	++	+++	++
Clinic b4	24	+++	+	++	+++	++
Clinic b5	23	+++	+	++	+++	++
**Region C**						
Clinic c1	20	++	+	+	+++	+++
Clinic c2	12	+	+	+++		
Clinic c3	12	+	+	+++		
**Region D**						
Clinic d1	16	+	+++	+	+++	+++
Clinic d2	17	+	++	+	++	++
Clinic d3	10	+	+	+++		
Clinic d4	14	+	+	+++		+
Clinic d5	13	++	++	++	++	++
Clinic d6	17	++	++	++	++	++
Clinic d7	15	++	++	++	++	++
Clinic d8	17	++	++	++	++	++

+ low focus ++ medium focus +++high focus

#### Mechanism 1: Focusing on the provider: “Improving the way clinic teams work”

Clinics guided by this mechanism centered on improving their workflow, involving three main strategies: improving team processes, changing the design of the delivery of services, and using patient registries to track performance cards and reminders.

Clinics aimed to improve team processes by allocating tasks and creating a shared team responsibility for improving processes of care. The main strategy all target clinics used was weekly team meetings to discuss ways for improving their performance on the QUIDS indicators and to perform consultations regarding care management of complex chronic patients. During each meeting, the team reviewed medical records and devised patient-centered interventions. Team members viewed the implementation of routine meetings not only as a new strategy to improve care but also as a chance to break traditional divisions of responsibilities, which often lead to working in silos (a1, a2, a3, a6, a10, b1, b3, b4, c1, d2, d5, d7, d8).

Clinics also implemented strategies aimed at improving accessibility of health care services, especially those relevant to the QUIDS indicators. For example, to increase the number of women obtaining mammography screening, a mobile screening unit was transported to large clinics that participated in the program (a1, a6, a8, c1, d2, d7, d8). Medium-sized clinics and those close to medical centers provided a shuttle from clinic to hospital for women who needed mammography or other screening tests (b1, b2, b3, a2, a8, a10, c2, d1). Another widely adopted strategy was the creation of shared-care schemes in which an ophthalmologist rotated between primary care clinics, performing fundus tests for diabetic patients (a1, a2, a5, a6, b1, b2, b5, d6).

Using Clalit’s computerized patient registry, all 26 clinics monitored their performance and identified patients for targeted intervention in all seven chosen indicators. This included identification of patients in need of screening tests or patients with poor diabetes or hypertension control. For example, clinics used the registry to identify patients in need of occult blood tests, administered testing kits to target populations, and followed up on returned screening kits.

In cases where management identified gaps in clinic teams’ knowledge when working with the organization’s computerized registry, technical support personnel were recruited to guide the teams in these processes (a6, a8, b5, c2, d2, d5, d8). For example, region A identified effective use of patient registry as a problem in all targeted clinics and set up a ‘QI school’ to train teams to retrieve data from the system, analyze performance, and devise intervention strategies.

#### Mechanism 2: Focusing on the patient: “Tailoring services to patients and their community”

Clinics for which the driving mechanism was the ‘*focus on the patient’* performed activities such as cultural adaption of services and identified a need to better tailor their services to the unique needs of the populations they served. They employed a wide range of patient-education interventions aimed at improving chronic-disease care. For example, clinic c1 created a culturally tailored cooking class for Arab minority diabetic patients as well as a workshop led by a nurse and a religious leader on the importance of maintaining a healthy lifestyle. Through the use of religious scripts, patients were educated about how Islam views dietary habits and exercise. Clinic a1 publicized the importance of early cancer screening in the local newspaper, including testimonials of colon cancer patients who are community members. Clinic d1 created a pamphlet for ultra-orthodox Jewish mothers on the importance of iron supplements for infants, introducing culturally tailored messages. Clinic d5 created a tailored pamphlet on the consumption of honey and its effect on diabetes control, as honey products are commonly used in traditional sweets of Arab populations.

Most target clinics implemented health-promotion and patient-education activities in the community through lectures and health-promotion fairs held in local community centers (a1, a3, a6, b1, b2, b4, c1, d1, d2, d6). Additional activities included a weekly spot on local radio to enhance knowledge and promote activities such as the importance of vaccination (Clinic a8), and referral of ultra-orthodox Jewish women to screening services at a hospital that provides care to ultra-orthodox religious populations, despite the clinic’s closer proximity to another hospital (d1). Several clinics included flu vaccinations as part of the vaccination regimen administered to Muslims embarking on their religious pilgrimage to Mecca (a8, b3, d2, d5). Others focused on educating religious leaders and administering information on the importance of vaccination during prayer times (a8, a10, b3, b5, d8).Strategies focusing on the patient also included tailoring care plans for control of diabetes or hypertension. Clinic teams crafted care plans taking into account patients unique cultural practices. For example, in many of the clinics serving diabetic Muslim patients, care plans took into account the unique practice of the month of Ramadan and how to account for control when patients fast daily and break the fast with traditional sweets. Clinic b2, for example, implemented a unique patient education and care plan around hypertension Bedouin patients to address the issue of traditional coffee drinking.

#### Outcome: Clinics’ QUIDS score

Table 3 presents QUIDS scores for all clinics at baseline and 3-year follow-up. Clinics varied in the extent of the gap measured at baseline: 7 clinics had a relatively small gap (≤0.06 QUIDS points), 7 had an intermediate gap (0.06–0.09 QUIDS points), and 12 had a large gap (>0.09 QUIDS points). At follow-up, 12 clinics had closed more than 80% of their initial gap, and 7 had decreased their gap by less than a third or even slightly increased it.

**Table 3:** Outcomes: Change in QUIDS score of target clinics.

	Gap in QUIDS Score 2009	Gap in QUIDS Score 2012	Disparity Reduction (%)*
**Region A**			
Clinic a1	0.186	0.088	-52.9
Clinic a2	0.060	0.030	-100.0
Clinic a3	0.068	0.045	-34.3
Clinic a4	0.053	0.059	10.5
Clinic a5	0.244	0.072	-70.6
Clinic a6	0.110	0.100	-8.7
Clinic a7	0.042	-0.124	-100.0
Clinic a8	0.147	-0.038	-100.0
Clinic a9	0.078	0.032	-58.9
Clinic a10	0.133	-0.072	-100.0
**Region B**			
Clinic b1	0.089	0.006	-92.9
Clinic b2	0.079	0.002	-98.0
Clinic b3	0.008	0.008	3.2
Clinic b4	0.034	0.006	-81.2
Clinic b5	0.059	0.037	-37.6
**Region C**			
Clinic c1	0.092	0.016	-83.1
Clinic c2	0.061	0.025	-58.8
Clinic c3	0.015	-0.009	-100.0
**Region D**			
Clinic d1	0.113	0.013	-88.8
Clinic d2	0.157	0.025	-83.9
Clinic d3	0.074	0.006	-91.5
Clinic d4	0.065	0.085	31.6
Clinic d5	0.120	0.082	-31.8
Clinic d6	0.144	0.096	-32.9
Clinic d7	0.122	0.093	-23.6
Clinic d8	0.185	0.100	-46.3

*Percent reduction of gap was truncated at 100, i.e., clinics in which the follow-up score exceeded the regional average received a 100% closure of the gap score.

### Phase III: Constructing context-mechanism-outcome configurations

We first categorized clinics based on the degree to which attaining the outcome (i.e., closing the gap in QUIDs score) was challenging in terms of the baseline score. Five CMO configurations were identified based on size of the initial gap, degree of attainment of the disparity-reduction goal, and types of context and mechanisms employed. Two configurations were observed for clinics with a small gap to minimize, and three were identified for target clinics with a large gap (Table 4).

**Table 4:** Context-mechanism-outcome configurations of target clinics.

Category	Context	Mechanisms	Interventions Implemented	Outcome: Reducing the Gap	Target Clinics
Clinics with a small gap to minimize: CMO A	High clinic density, High clinic–subregional headquarters density, High perceived TE.	A focus on changes to both provider and patient	A high number of interventions implemented in all areas of change	Clinics reduced the gap	a2, b3, b4, b5
Clinics with a small gap to minimize: CMO B	Medium or low clinic density, Low clinic–subregional headquarter density, Medium or low perceived TE.	A focus on the Provider	A small number of interventions centering on IT, mostly instigated by management	Clinics were not able to reduce the gap significantly	a3, a4, c2, c3, d3, d4
Clinics with a large gap to minimize: CMO C	High clinic density, medium-high subregional management density Medium-High TE.	A focus on the patient	Medium-High number of interventions focused on adapting services to patients as well as developing community linkages	Clinics reduced the gap significantly	a8, a10, b1, b2, c1, d1, d2
Clinics with a large gap to minimize: CMO D	Medium clinic density, High Clinic–subregional management density. High perceived TE	A focus on the provider	A small number of interventions implemented mostly instigated by management	Clinics reduced the gap significantly	a5, a7, a9
Clinics with a large gap to minimize: CMO E	Medium clinic density, Medium clinic–subregional headquarters density, low-medium-perceived TE.	A focus on changes to both provider and patient	Medium number of interventions	Clinics were not able to reduce the gap significantly	a1, a6, d5, d6, d7, d8

TE = Team effectiveness.

*CMO A*. These clinics had a small gap at baseline and succeeded in minimizing that gap. They had highly motivated teams with strong ties among them, employed many interventions to improve care management, and tailored services to the specific needs of their patient populations (a2, b3, b4, b5) (Table 4).

*CMO B*. These clinics did not attain the QI disparity-reduction goal even though they had only a small gap. This was related to each clinic’s inner contextual characteristics of low clinic density, low clinic subregional density, and low perceived TE. Team members of these clinics mentioned internal problems of working together as a team, as well as a lack of organizational support. Interventions were few, focused on use of the patient registry, such as scheduled reminders, and were mostly instigated by subregional headquarters (a3, a4, c2, c3, d3, d4).

*CMO C*. These clinics were characterized by a large gap at baseline, which they closed. All clinics reported high levels of clinic density, medium to high clinic subregional density, and perceived themselves as capable of implementing the program. They identified the unique needs of the populations they served and tailored all their interventions, creating a better fit between services provided and their patients’ unique needs (a8, a10, b1, b2, c1, d1, d2).

*CMO D*. These clinics were characterized not only as having an initial large gap to minimize but also as having low clinic density and low perceived TE. Nonetheless, they differed from CMO B clinics due to their high density with subregional headquarters. Although few interventions were implemented, management’s focus and support of clinic teams during implementation of the program by holding weekly visits and meeting with clinic staff, for example, helped attain the desired goal (a5, a7, a9).

*CMO E*. These clinics had a large initial gap to close, and reported internal structural difficulties. All had low-medium density and perceived TE. Clinic-subregional density scores were also intermediary ranked. In this group, as compared to clinics in CMO B, clinic teams worked not only to devise a strategy for improving teamwork, but also to tailor services to meet patients’ special needs (a1, a6, d5, d6, d7, d8). These interventions, however, did not result in reduced disparity gaps.

To summarize, clinics that managed to reduce the gap and had a small initial gap to close, had high clinic density, high clinic–subregional headquarters density, and high perceived TE, these clinics implemented a high number of interventions in all areas of change focusing on changes to both provider and patient. These, however, constituted only a relatively small number of clinics (4 of 10 with a small initial gap). Clinics that managed to reduce the gap and had a large initial gap to close, either had (1) a High clinic density, medium-high subregional management density Medium-High TE and a medium-high number of interventions, yet they focused on adapting services to patients as well as developing community linkages, or (2) had Medium clinic density, high clinic–subregional management density, and high perceived TE, yet a small number of interventions implemented which were mostly instigated by management. Nonetheless, of all clinics with a large initial gap, most (10/16) managed to significantly close the gap.

## Discussion

Our study focused on identifying mid-range theories and the causal linkages between context, mechanisms, and outcomes achieved in a disparity-reduction program. The first phase of the study identified QI as the program theory underpinning the disparity reduction efforts and guiding the ideas and assumptions of teams implementing the program. Understanding that QI is the guiding framework enabled us to identify the mechanisms and contexts in which the program was implemented, as well as the boundaries in which teams operated, i.e., their ability to benefit from the structure and framework of QI as well as their need to tailor their approach and tools for achieving disparity reduction given the limitations of QI. The QI approach benefited the teams by directing their actions through highlighting the measures that required improvement in their clinic's population. However, as quality indicators focus on specific elements of care, rather than how care should be delivered [48] teams were tasked with developing and culturally adapting care strategies to meet their specific population's needs. The advantages and limitations of the QI approach are reflected in the two broad mechanisms identified: (a) A focus on the provider, especially improving teamwork and service delivery design, and (b) A focus on the patient and tailoring programs to meet patient and community needs. These mechanisms explain the reasoning, actions and responses of teams to organizational program focused on achieving quality improvement disparity reduction [20].

Target clinics focused on improving how teams operate through organizational redesign as well as effective use of performance data. CMO configurations suggest that improvement of internal organizational processes, such as training clinic teams to measure and use clinical indicators, were a necessary step toward achieving disparity reduction. Similarly, Thorlby and colleagues (2008) [49] found that information systems were viewed as essential components of disparity-reduction efforts, as they allowed not only identification of existing inequities but tailoring of interventions accordingly. Nadeem and colleagues (2013) [50], in their systematic review of QI collaboratives, also report that components such as data reporting, training providers in QI techniques, and leadership involvement had the greatest impact on QI program success. Similarly, we found that all successful clinics had close ties with subregional management. Birken and colleagues (2012), [51] state that middle managers contribute to the attainment of success in QI programs by creating a bridge between the contextual factors affecting the implementation in each of their subordinate units and managing the demands of top management.

Our findings on the implementation of patient-centered interventions receive credence from a review spanning 30 years of QI disparity-reduction efforts [1], which showed that most interventions were targeted toward improving patient-centered care. Methods to trigger behavioral change centered on patient education. However, patient education was found to be a ‘necessary but not sufficient’ step. The authors conclude that organizations need to address not only the patient but the provider through interventions focused on the care-delivery system. Fiscella and Epstein (2008) [52] note that during a 15-minute visit it is impossible for physicians to properly address the needs of disadvantaged patients and that most implemented interventions need to extend the scope of clinic visits. They suggest the use of primary health-care teams centered around patients’ needs and working together as an inter-professional care-management team, as a framework for action. Similarly, we found that the implementation of weekly joint consultations for chronic-disease patients with complex care needs was associated with the attainment of disparity-reduction goals.

We also found that a focus on addressing patients’ unique sociocultural needs was associated with success. Chin and colleagues [53] found that culturally tailored interventions targeting different causes of disparities hold the most promise in effectively reducing disparities. Betancourt and colleagues (2014) [47] highlight that successful disparity-reduction efforts do not center only on language barriers but address the values, beliefs, and behaviors of cultural groups. Clinics that implemented culturally tailored interventions recognizing the unique social values and beliefs of communities, such as working together with religious leaders on the importance of lifestyle and adherence to diabetes treatment, reduced their gap and sustained this reduction over time.

Our study has several limitations. We recognize the possibility of recall bias as a threat to the validity of theory-informed data collection and note that the RE framework aims to assess the program during its implementation, refining proposed theories through participant feedback [23]. We tried to address this limitation by conducting a member check to allow respondents the opportunity to refine and give feedback on the strategies observed. A related limitation is that our data were derived from 26 target clinics and not all 55 clinics included in the project. Nonetheless, as presented in the methods section, the study clinics represented a wide range of characteristics that reflect the type of population and clinics included in the larger project. Furthermore, conducting interviews with a wide range of personnel directly involved in implementing the disparity-reduction program across a variety of urban and rural settings, and in various population groups, enabled us to examine whether the program succeeded across a wide spectrum of sites [54].

## Conclusion

Our study shows that theorizing the logic of QI disparity-reduction programs and understanding how it relates to both the individuals involved and the context in which programs are implemented may help us understand not only whether a program works but also how it works, and under what circumstances disparity reduction can be achieved [55]. Our study identifies the context in which QI disparity-reduction strategies should be applied by linking identified mechanisms with program activities and observed outcomes. We show that clinics that achieved disparity reduction had highly dense teams, close ties with subregional management, and identified the unique needs of the populations they serve.

## Supporting information

S1 File Questionnaire adapted from Shortell et al.docx. (PDF)
