# Supplementary material for: Evaluating an organization-wide disparity reduction program: Understanding what works for whom and why
Source: PLoS One. 2018 Mar 14;13(3):e0193179. doi: 10.1371/journal.pone.0193179 (PMC5851553; doi:10.1371/journal.pone.0193179)
Supplement: S1 File — (PDF) [file pone.0193179.s001.pdf]

Questionnaire adapted from Shortell et al (2004) and Zohar et al (2008):

	Disagree				Agree
The team got all the information we needed to plan our work	1	2	3	4	5
Our team had the authority to manage its work pretty much the way members wanted to					
There was a great deal of room for initiative and judgment in the work that we did					
The participants on our team are “process owners” for this work, that is, they have substantial influence in managing care and influencing others to make improvements in care					
When our team did not know something it needed to know to do its work, there were people available to teach or help					
Almost all of our change processes were wider than just one-focus improvement process such as patient education alone					
Out team chose additional quality improvement measures to those chosen by management					
Our team used measures regulary basis to assess and decide on change					
After we have completed a change, team members are excellent in reflecting and learning from the results					
Members of our team use information from our change cycles to design new tests of change					
In making changes, our team was able to easily adapt change ideas to match the needs of our organization					
Our team has enough knowledge and skill to get the work done well					
Project team members agreed on the project’s overall goals and how to achieve improvement					
Most team members got a chance to participate in decision-making processes					
Certain individuals in this group had special skills and knowledge that the rest of us counted on					
All team members contributed and were involved in the process					
The project’s goals were understood by all project team members					
This organization makes sure people have the skills and knowledge to work in teams					
A team that does a good job in this organization does not get any special rewards or recognition					
Senior management in the organization strongly supports our work					

Senior management regularly reviews our progress in making change					
There is involvement of community members in our change activities					
The team received sufficient resources to implement their quality improvement initiatives					

Please circle your position in the clinic:

1. Medical Director 2. Nursing Dirctor 3. Administrative Director 4. Other:_________

Of the following people in the organization, to what extent do you converse regularly about quality improvement initiatives in your clinic:

	rarely				often
**In the clinic:**					
Clinic/medical director	1	2	3	4	5
Nursing Director	1	2	3	4	5
Administrative Director	1	2	3	4	5
Other:_______________	1	2	3	4	5
**In the sub-regional Headquarters**					
Medical director	1	2	3	4	5
Nursing director	1	2	3	4	5
Administrative director	1	2	3	4	5
Quality Improvement coordinator	1	2	3	4	5
Other:________________	1	2	3	4	5
**In the Regional Headquarters**					
Medical director	1	2	3	4	5
Nursing director	1	2	3	4	5
Administrative director	1	2	3	4	5
Quality Improvement coordinator	1	2	3	4	5
Other:________________	1	2	3	4	5
